# Use of a Diabetes Self-Assessment Score to Predict Nonalcoholic Fatty Liver Disease and Nonalcoholic Steatohepatitis

**DOI:** 10.1097/MD.0000000000001103

**Published:** 2015-07-13

**Authors:** Gyuri Kim, Yong-ho Lee, Young Min Park, Jungghi Kim, Heesuk Kim, Byung-Wan Lee, Eun Seok Kang, Bong-Soo Cha, Hyun Chul Lee, Dae Jung Kim

**Affiliations:** From the Department of Internal Medicine, Yonsei University College of Medicine, Seoul, Republic of Korea (GK, Y-hL, B-WL, ESK, B-SC, HCL); Department of Medicine, Yonsei University College of Medicine, Seoul, Republic of Korea (GK, Y-hL, B-WL, ESK, B-SC, HCL), Graduate School, Yonsei University College of Medicine, Seoul, Republic of Korea (GK, Y-hL, B-WL, ESK, B-SC, HCL); Yonsei University College of Medicine, Seoul, Republic of Korea (JK, HK); Department of Family Medicine, National Health Insurance Corporation Ilsan Hospital, Goyang, Republic of Korea (YMP); Department of Endocrinology and Metabolism, Ajou University School of Medicine, Suwon, Republic of Korea (DJK).

## Abstract

Nonalcoholic fatty liver disease (NAFLD) and nonalcoholic steatohepatitis (NASH) are strongly associated with obesity, insulin resistance, and type 2 diabetes. We recently developed and validated a self-assessment score in the Korean population to identify people at high risk for diabetes. The objective of this study was to evaluate whether the self-assessment risk score for diabetes can also be used to screen for the presence of NAFLD or NASH.

The study population included 15,676 subjects (8313 men and 7363 women) over 20 years old who visited the National Health Insurance Service Ilsan Hospital in Korea between 2008 and 2010. Anthropometric, clinical, and laboratory data were analyzed during regular health checkups. Fatty liver disease was diagnosed using ultrasound, discrimination capability was assessed based on the area under the receiver operating characteristic curve (AUC), and evaluation measures, including sensitivity and specificity, were calculated. Multiple logistic analyses were also performed.

We calculated a self-assessed risk score for diabetes (range: 0–11), and a cutoff of ≥5 identified 60% (50%) of men (women) at high risk for NAFLD, reflecting a sensitivity of 79% (85%), a specificity of 60% (66%), a positive predictive value (PPV) of 68% (51%), and a negative predictive value (NPV) of 73% (91%), with an AUC of 0.75 (0.82) for men (women). A cutoff point of ≥6 identified 43% (31%) of men (women) at high risk for NASH, reflecting a sensitivity of 80% (86%), a specificity of 64% (75%), a PPV of 30% (28%), and a NPV of 94% (98%), with an AUC of 0.77 (0.86) for men (women). The odds ratios that a 1-point increase in the diabetes risk scores would be associated with an increased risk for NAFLD and NASH were 1.20 [95% confidence interval (CI): 1.16–1.25] and 1.57 (95% CI: 1.49–1.65), respectively, in men, and 1.28 (95% CI: 1.21–1.34) and 1.89 (95% CI: 1.73–2.07), respectively, in women.

The present study indicates that our self-assessment risk score for diabetes could be an effective primary screening tool for the presence of NAFLD or NASH.

## Abstract 2

Supplemental digital content is available in the text

## INTRODUCTION

Obesity, one of the most rapidly increasing health problems in the world,^1^ is associated with morbidity and mortality, and contributes to the burden placed on society by chronic public health conditions.^2,3^ Obesity has been identified as a leading contributor to insulin resistance,^4,5^ and many previous studies have shown that insulin resistance is strongly associated with the development of type 2 diabetes (T2D) and nonalcoholic fatty liver disease (NAFLD).^6–9^ Furthermore, nonalcoholic steatohepatitis (NASH) has also been associated with insulin resistance syndrome, including obesity, T2D, and hypertriglyceridemia.^10,11^ NAFLD can transition to NASH as a function of the progression of hepatic damage, inflammation, and fibrosis, and NASH can develop into cirrhosis, and ultimately, hepatic cancer.^12–14^ In view of the impact of the various complications of NAFLD and NASH, the early diagnosis of these conditions is an important clinical issue facing public healthcare systems. However, NAFLD and NASH are difficult to diagnose in clinical practice due to the need for an invasive procedure, that is, a liver biopsy. Although several simple, noninvasive clinical scoring systems for predicting NAFLD or NASH have been proposed, they are not widely used because they involve a blood assay and complex scoring formulas.^15–18^ Also, recently noninvasive tools such as abdominal ultrasonography or transient elastography for accessing liver steatosis or fibrosis have used yet, further confirmative data are warranted and costs are high.^19,20^

We recently developed and validated a self-assessment tool to assess diabetes risk that does not require blood assays or mathematical calculations.^21^ The model includes age, family history of diabetes, hypertension, waist circumference, smoking status, and daily alcohol consumption, but does not incorporate laboratory parameters. A recent study found that this model was also validated as a predictor of prediabetes as well as of T2D.^22^ Therefore, given that insulin resistance is common to both NAFLD and NASH, the aim of this study was to investigate and validate this diabetes risk score for the prediction of these conditions in the adult population.

## METHODS

### The Study Population

Between 2008 and 2010, 18,765 individuals aged over 20 years who visited the National Healthcare Insurance Ilsan Medical Center (NHIMC) in Ilsan, South Korea for comprehensive health examinations were included on this study. Exclusion criteria were as follows: history of alcohol abuse as indicated by weekly alcohol consumption >140 g for males and >70 g/week for females (N = 778); any etiological markers for chronic liver disease, including positive serologic markers for hepatitis B virus (N = 752), hepatitis C virus (N = 130), or human immunodeficiency virus (N = 1); presence of thyroid disease, including hyperthyroidism, hypothyroidism, or thyroid hormone replacement therapy (N = 118); abnormal ultrasonographic liver findings (ie, suspected hepatocellular carcinoma, hepatic mass, or signs of *Clonorchis sinensis*) (N = 971); and/or absence of questionnaire data or anthropometric measurements (N = 1135). Data from a total of 15,676 individuals were analyzed in this study.

The protocol of this study was approved by the Institutional Review Board of the Ilsan Hospital, and written informed consent for this study was not required because researchers accessed only the database for purposes of analysis, and personal information was not used.

### Data and Measurements

All participants provided data regarding their demographic characteristics; personal and family medical history; social habits, including smoking and alcohol consumption; physical activity; and use of medication at the time of their clinical consultation. Body mass index (BMI) was calculated by weight (kg)/height (m^2^). Waist circumference was obtained at the minimal point between the lowest rib and the upper iliac crest after normal expiration. Laboratory parameters were also measured after overnight fasting. The product of fasting triglycerides and glucose levels (TyG), a surrogate for identifying insulin resistance, was estimated as the Ln[fasting triglycerides (mg/dL) × fasting glucose (mg/dL)/2].^23^ Subjects were classified as having diabetes if they were taking an oral hypoglycemic agent or insulin, or had been previously diagnosed with diabetes by a healthcare professional. Impaired fasting glucose was defined as a fasting plasma glucose level of 100–125 mg/dL as per the 2011 revision of the American Diabetes Association (ADA) guidelines.^24^ Hypertension was defined as taking antihypertensive medication or having been diagnosed with this condition by a physician. Individuals were categorized with regard to smoking status according to self-reports as follows: never, ex-smoker, and current smoker. Daily alcohol consumption was quantified by types of beverages, frequency of drinking, and average amount of alcohol consumed on each occasion, as previously described.^21^ After excluding subjects with excessive alcohol intake, alcohol consumption was classified based on the daily amount of alcohol consumption: none, <1, 1–4.9, or ≥5 drinks daily. Exercise status was assessed by self-reported questionnaires that included questions about the duration, frequency, and types of exercise. Regular exercise was then defined as engaging in physical activity for at least 30 minutes at least twice weekly.

All subjects underwent abdominal ultrasonography (Sonoline Antares MSC 2704 AB; Siemens Medical Solutions, Issaquah, WA) performed by trained radiologists who were blind to the patients’ clinical and laboratory data using a 3.5-MHz transducer. The severity of the fatty liver was classified into 3 grades (mild, moderate, and severe) according to standard criteria.^25^ We also recorded the absence or presence of fatty liver disease.

Due to its invasiveness, a liver biopsy was not performed, and liver fibrosis was identified using previously reported NAFLD fibrosis scores based on the following formula: –1.675 + 0.037 × age (years) + 0.094 × BMI (kg/m^2^) + 1.13 × impaired fasting glucose (IFG)/diabetes (yes = 1, no = 0) + 0.99 × aspartate transaminase (AST)/alanine transaminase (ALT) ratio – 0.013 × platelet (×10^9^/L) – 0.66 × albumin (g/dL).^16^ Liver fibrosis was classified based on NAFLD fibrosis scores: –1.445 to 0.676 was coded as indeterminate fibrosis, and >0.676 was coded as advanced fibrosis.^16^

The Korean Diabetes Score (KDS) questionnaire was administered to individuals by registered interviewers. As previously described,^21^ points were accrued for the following parameters: age: <35 years was scored as 0, 35–44 years as 2, and ≥45 years as 3; waist circumference: percentiles 1–50 were scored as 0, percentiles 51–74 as 2, and 75th percentile and above as 3; daily alcohol intake: <1 drink was scored as 0, 1–4.9 drinks as 1, and ≥5 drinks as 2; and family history of diabetes, history of hypertension, and smoking status were scored as 0 or 1.

### Statistical Analyses

All continuous variables are presented as means ± standard deviations (SDs), and categorical variables are expressed as frequencies with percentages. Differences were analyzed using analysis of variance (ANOVA) for continuous variables and Chi-square tests for categorical variables. The ability of the KDS to predict NAFLD and NASH were evaluated by receiver operating characteristic (ROC) curves. The area under the receiver operating characteristic curve (AUC) was used to compare the accuracy of tests in terms of their ability to make relevant distinctions. In addition, we compared the performance of the KDS using the following screening models for NAFLD or NASH: FIB-4, [age × AST (IU/L)/platelet count (×10^9^/L) × ALT (IU/L)^1/2^]^18^; APRI (AST-to-platelet ratio index), [(AST/upper limit of normal)/platelet count (10^9^/L) × 100]^17^; and AAR (AST/ALT ratio).^26^ Data on sensitivity, specificity, positive predictive value (PPV), negative predictive value (NPV), likelihood ratios (LRs; positive and negative), the Youden Index, and the AUC were analyzed to evaluate the measures.^21,27,28^ The odds ratios (ORs) and 95% confidence intervals (CIs) for the factors associated with the prevalence of NAFLD or NASH were calculated using multiple logistic regression analysis. A *P* < 0.05 was considered statistically significant. Statistical analyses were performed using SAS version 9.2 (SAS Institute, Cary, NC), SPSS version 20.0 (SPSS, Chicago, IL), and MedCalc (version 13.1; http://medcalc.software.informer.com/13.1/).

## RESULTS

### Baseline Characteristics of the Study Population

The baseline clinical and laboratory characteristics of the study population are shown in Table 1 according to the fatty liver condition(s) identified by abdominal ultrasonography and NAFLD fibrosis scores. According to our data, 58.8% (9221) of the subjects with a normal liver showed no evidence of a fatty liver on ultrasonography, and 28.0% (4386) of the subjects with simple steatosis showed a fatty liver on ultrasonography and the absence of fibrosis according to NAFLD fibrosis scores. Subjects with steatohepatitis, a fatty liver, and intermediate or advanced fibrosis, as assessed by NAFLD fibrosis scores, accounted for 13.2% (2069) of the sample. Subjects with steatosis or steatohepatitis tended to be older, more obese, smoke more at the time of evaluation, more likely to drink alcohol on a daily basis, and to have higher laboratory values for metabolic factors compared to those without steatosis. Males and those with hypertension, diabetes, or abnormal fasting glucose levels were more likely to have steatosis or steatohepatitis.

**TABLE 1:**
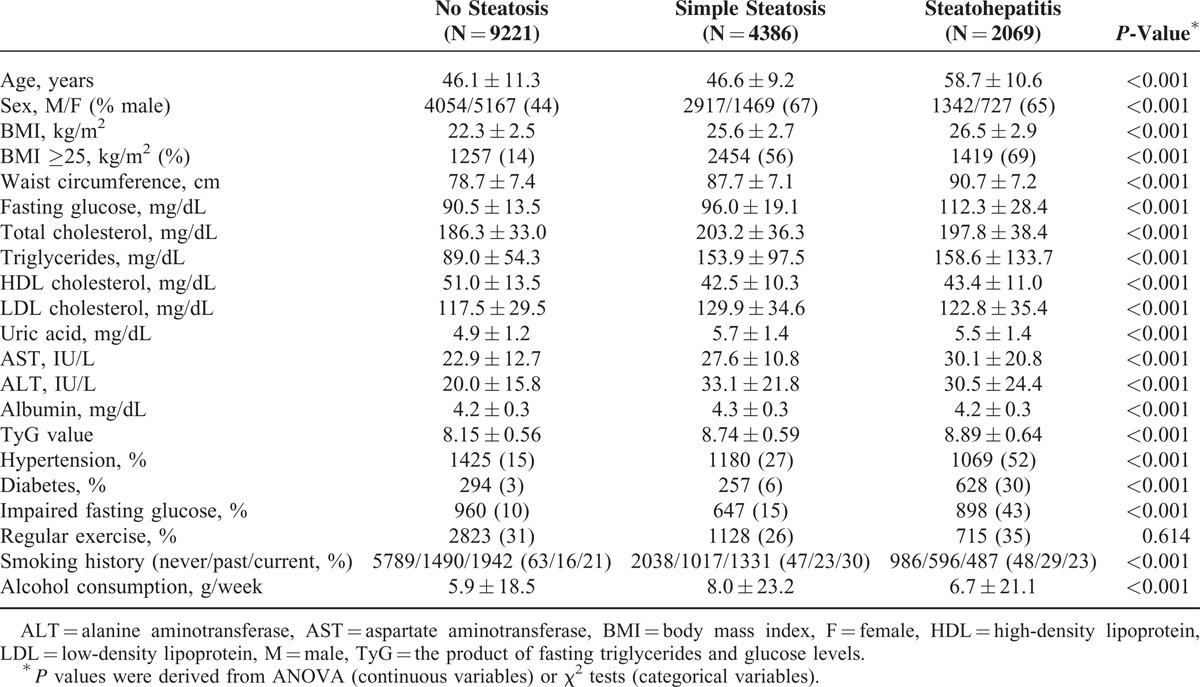
Baseline Characteristics of the Subjects

### Predictive Power of the Korean Diabetes Score (KDS) for NAFLD and NASH

**FIGURE 1:**
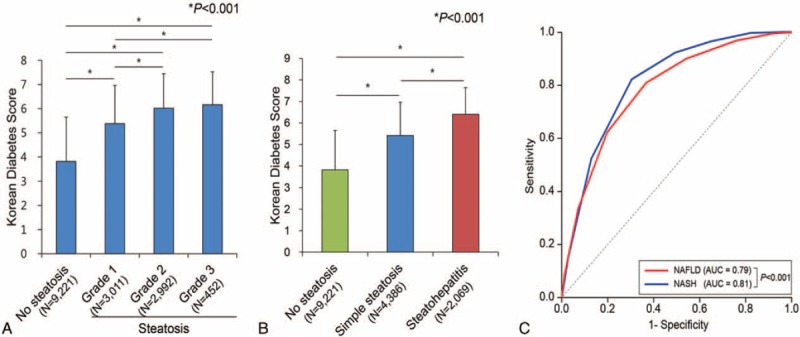
Relationship between the Korean Diabetes Score and NAFLD or NASH. (A) Average KDS values according to fatty liver grade, as determined by hepatic ultrasound. (B) Average KDS values according to fatty liver condition, as determined by NAFLD fibrosis scores and hepatic ultrasound. Subjects with a normal liver (N = 9221) showed no evidence of a fatty liver on ultrasonography. Simple steatosis (N = 4386) indicates an ultrasonographically defined fatty liver in the absence of advanced fibrosis, according to NAFLD fibrosis scores. Steatohepatitis (N = 2069) includes subjects with fatty liver with intermediate or advanced fibrosis, according to NAFLD fibrosis scores. ^∗^All *P* values are <0.001. (C) ROC curves with KDS values for the prediction of NAFLD or NASH in the study population. KDS = Korean Diabetes Score, NAFLD = nonalcoholic fatty liver disease, NASH = nonalcoholic steatohepatitis, ROC = receiver operating characteristics.

Figure 1A presents the average values of the KDS according to liver steatosis status as assessed by abdominal ultrasonography. The average KDS value increased significantly as the grade of liver steatosis increased. Additionally, according to ultrasonagraphic data on fatty liver condition and NAFLD fibrosis scores, simple steatosis and steatohepatitis showed significant tendencies toward associations with increased KDS values (Figure 1B). Figure 1C presents the ROC curve for the prediction of NAFLD or NASH in the study population. The areas under the ROC curve were 0.79 (95% CI: 0.78–0.79; *P* < 0.001) and 0.81 (95% CI: 0.81–0.82; *P* < 0.001) for NAFLD and NASH, respectively.

We investigated the diagnostic characteristics associated with the use different KDS cutoff values for the prediction of NAFLD or NASH. Use of a KDS cutoff value of 5 points to predict NAFLD was associated with the highest values respectively for males and females with regard to the following: the Youden Index, 39 and 51; sensitivity, 79% and 85%; specificity, 60% and 66%; and AUC, 0.75 and 0.82 (Table 2). A KDS cutoff value of 6 points was selected for the prediction of NASH, as it was associated with a higher overall level of test accuracy according to the Youden Index; it identified 37% of subjects at high risk for NASH and yielded a sensitivity of 80% and 86%, a specificity of 64% and 75%, and AUC values of 0.77 and 0.86 for males and females, respectively (Table 3).

**TABLE 2:**
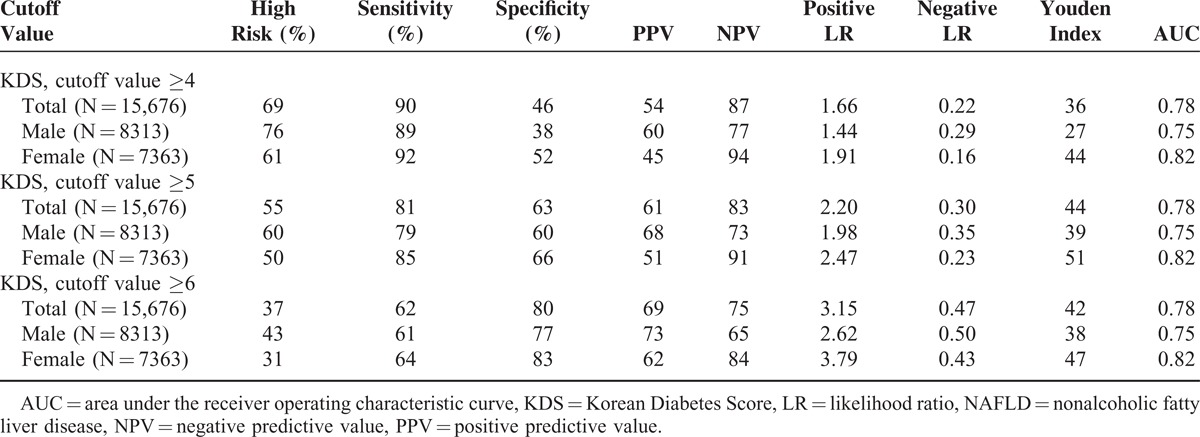
Predictive Power of the Korean Diabetes Score for NAFLD

**TABLE 3:**
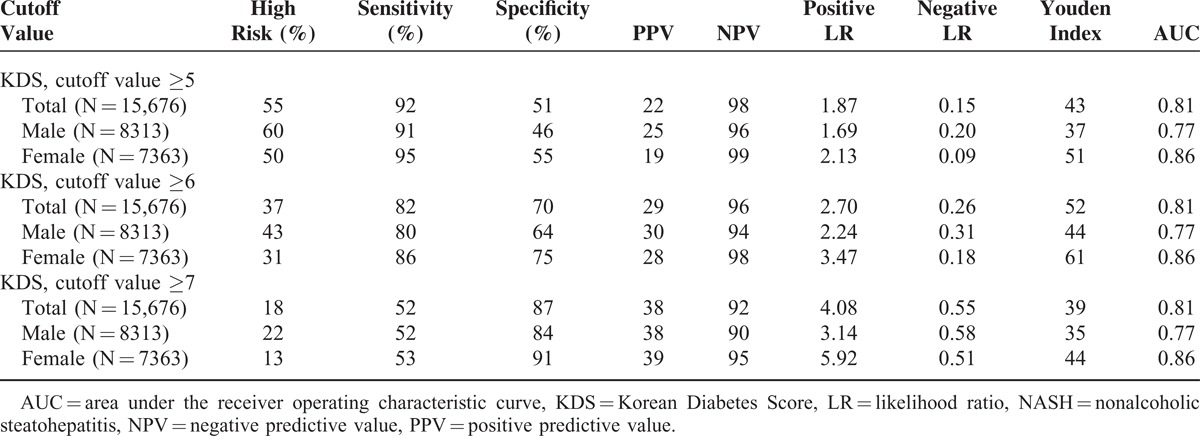
Predictive Power of the Korean Diabetes Score for NASH

**FIGURE 2:**
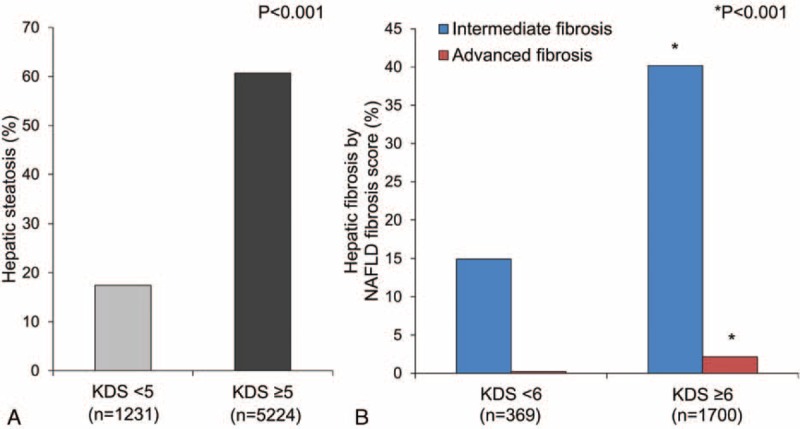
Proportions of subjects with hepatic steatosis (A) and fibrosis according to NAFLD fibrosis score (B) according to the Korean Diabetes Score. KDS = Korean Diabetes Score, NAFLD = nonalcoholic fatty liver disease.

Figure 2A and B present the ability of cutoff values of 5 and 6 on the KDS to identify subjects who had been diagnosed with hepatic steatosis by abdominal ultrasonography and with fibrosis by NAFLD fibrosis scores, respectively. Figure 2A shows that 60.7% subjects with KDS values higher than 5 points (17.4% of subjects with KDS values lower than 5, *P* < 0.001) met the criteria for hepatic steatosis. The prevalence of intermediate and advanced fibrosis (40.2% and 2.1%, respectively; *P* < 0.001) in subjects with KDS values higher than 6 points was significantly higher than that in those with KDS values lower than 6 points (14.9% and 0.2%, respectively, *P* < 0.001; Figure 2B). We also applied the KDS cutoff value of 6 points to other noninvasive liver fibrosis scoring systems, such as those for FIB-4, APRI, and AAR, to compare its predictive power for liver fibrosis with that of the NAFLD fibrosis scoring system because we did not perform invasive liver biopsy procedures in this study. The NAFLD fibrosis scoring system, FIB-4, APRI, and AAR identified statistically significant different proportions of subjects with hepatic fibrosis when a KDS value higher than 6 points was used (Supplementary Figure 1, http://links.lww.com/MD/A321).

### Use of the Korean Diabetes Score and Other Parameters to Predict NAFLD or NASH

Multiple logistic regression analyses were performed on KDS values and clinical and laboratory parameters to investigate important predictors of NAFLD and NASH (Table 4). In the basic model (Model 1), the ORs that a 1-point increase in KDS values would be associated with an increased risk for NAFLD were 1.73 (95% CI: 1.68–1.78) in men and 2.18 (95% CI: 2.09–2.27) in women. In the comprehensive model (Model 2), both KDS values and other parameters such as BMI, fasting glucose, high-density lipoprotein (HDL) cholesterol, low-density lipoprotein (LDL) cholesterol, uric acid, AST, ALT, and TyG value were independently associated with NAFLD. In term of the risk of NASH, every 1-point increase in KDS values was a strong predictor of the presence of this condition (OR: 1.92, 95% CI: 1.83–2.00 in men; OR: 2.81, 95% CI: 2.60–3.03 in women). In Model 2, KDS, BMI, fasting glucose, AST, ALT, and TyG value were significant independent predictors of NASH. The inclusion of KDS values and other parameters in this model produced more accurate results with higher values of AUC than Model 1, which incorporated only KDS values.

**TABLE 4:**
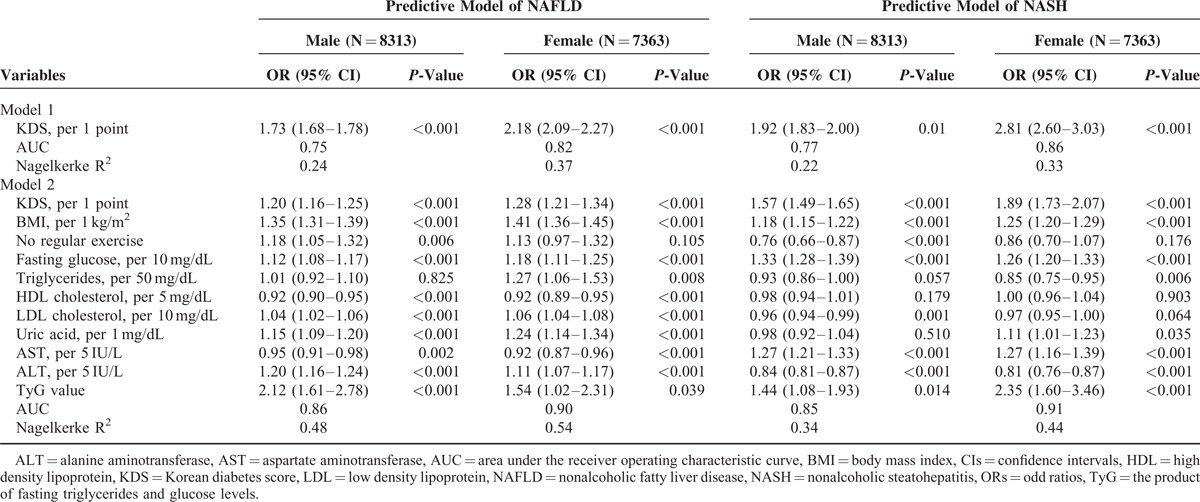
Odds Ratios (ORs) of Predictors of NAFLD and NASH

## DISCUSSION

In this study, we validated the KDS, a noninvasive tool for predicting NAFLD or NASH. The use of cutoff value of 5 points on the KDS was associated with a sensitivity and specificity of 81% and 63%, respectively whereas the use of the PPV and NPV to diagnosis or exclude NAFLD was associated with a respective sensitivity and specificity of 61% and 83%. Additionally, the good NPV of a score of 6 points with respect to NASH indicated that people at high risk should be referred to a specialist for early detection and intervention, including further laboratory studies and imaging tests.

NAFLD is defined by excessive fat accumulation in the form of cytoplasmic TG droplets, more than 5% of which are hepatocytes, in the liver.^29^ The pathogenesis of NAFLD is complex and remains poorly understood. Obesity and insulin resistance have been well established to increase the likelihood of developing NAFLD, and each of these factors is also characteristic of T2D.^6,7,30^ Given that one element of insulin resistance and such major contributing factors such as chronic inflammation are mediated by oxidative stress and hepatotoxic cytokines, T2D and NAFLD are closely associated based on their pathogenesis.^31–33^ Therefore, in this study, we aimed to investigate and validate the KDS, a useful self-report tool for assessing the risk of diabetes, with regard to its ability to predict NAFLD or NASH. A cutoff value of 5 points has been previously suggested to identify individuals at high risk of undiagnosed diabetes.^21^ Indeed, the present study confirmed the use of cutoff points of 5 and 6 for predicting NAFLD and NASH, respectively.

NAFLD is among the most rapidly increasing diseases worldwide, affecting one-third of adults in Western countries and rapidly increasing in Asian nations.^14^ NAFLD is often reversible and carries a good long-term prognosis, but it can progress to NASH, which is characterized by hepatocyte inflammation, and fibrosis, as well as dramatically increased risks for cirrhosis, and hepatocellular carcinoma.^8,12,14^ Therefore, an urgent need exists for the early detection of NAFLD or NASH so that individuals at high risk can access early interventions to prevent the progression of these conditions. In this context, the gold standard for diagnosing NAFLD or NASH has been a liver biopsy, but this method has drawbacks, including invasiveness, cost, sampling error, and intraobserver and interobserver variations. Indeed, performing liver biopsies on all at-risk individuals is not possible,^34^ and we need to develop noninvasive tools to replace liver biopsies. Therefore, several simple noninvasive clinical scoring systems, such as the NAFLD fibrosis score, FIB4, APRI, and AAR, have been developed to diagnose or exclude liver fibrosis.^15–18^ The KDS includes only 6 easily answerable questions regarding age, family history of diabetes, personal history of hypertension, waist circumference, smoking status, and alcohol consumption. Our examination of different fibrosis-prediction models (FIB4, APRI, and AAR) validated the use of a cutoff value of 6 on the KDS for predicting NASH. We confirmed that the KDS offers strong diagnostic power with regard to NAFLD or NASH without requiring laboratory assays.

In terms of the AUC, the KDS demonstrated a modest level of accuracy in predicting NAFLD (0.75 in males and 0.82 in females) and in predicting NASH (0.77 in males and 0.86 in females). The level of accuracy with which not only NAFLD (AUC of 0.86 in males and 0.90 in females) but also NASH (AUC of 0.85 in males and 0.91 in females) is identified could be increased by combining the KDS with other clinical and laboratory parameters. Of the laboratory parameters, BMI, fasting glucose levels, AST, ALT, and TyG value were independently associated with the prevalence of NASH in the entire sample. Furthermore, we found that a significantly higher risk of NASH than of NAFLD was associated with higher ORs for fasting glucose levels. Prolonged exposure to hyperglycemia can cause toxicity and induce apoptotic pathways in the liver^35^; thus, diabetes is more strongly associated with worse hepatic conditions such as NASH. In addition, the ORs for AST tended to increase and those for ALT tended to decrease with an increased risk of NASH. This finding is supported by a previous study showing that AAR values greater than 1 reflect a possible risk of the progression to NASH from NAFLD.^36^

Our study has several distinct strengths. First, from a practical perspective, the KDS is a simple and convenient tool with which laypersons can self-assess their risk of NAFLD or NASH without laboratory assays or expense. Second, the KDS was highly accurate in its prediction of NAFLD or NASH, and the PPV of KDS indicated its efficacy for screening subjects at high risk for NAFLD or NASH who should be referred for further laboratory studies and imaging and early intervention. The addition of laboratory parameters to the KDS will lead to more accurate predictions of NAFLD or NASH. NAFLD or NASH is estimated to increase 5-year direct and indirect medical costs by 26%.^37^ Therefore, economic and clinical benefit in the application of the KDS could be emphasized in terms of public health perspective. Finally, laypersons are able to identify and change the modifiable contributors to NAFLD and NASH based on the components of the KDS such as central obesity, smoking, and heavy alcohol consumption. Thus, individuals can modify their lifestyle to improve their health status.

The present study also has several potential limitations. First, a liver biopsy, the standard test for diagnosing NAFLD or NASH, was not performed. Because NAFLD was assessed by abdominal ultrasonography, which often does not identify early steatosis, the actual prevalence of NAFLD may have been underestimated. Additionally, we defined NASH indirectly, based on previously established noninvasive scoring systems such as the FIB4, APRI, and AAR. Next, this was a cross-sectional study on a Korean population, which limits our ability to generalize our conclusions in predicting the future development of NAFLD or NASH elsewhere. Future investigations using a longitudinal design are needed in this regard.

In conclusion, we investigated and validated the applicability of the KDS as a powerful tool for the prediction of NAFLD or NASH. As NAFLD, NASH, and T2D share common characteristics, such as obesity and insulin resistance, they can be ameliorated by early detection and intensive lifestyle modification. Therefore, the KDS could be used to alert individuals about their risk of developing NAFLD or NASH and might reduce the growing burden placed on society by these conditions.
